# Smoking Related Cancers and Loci at Chromosomes 15q25, 5p15, 6p22.1 and 6p21.33 in the Polish Population

**DOI:** 10.1371/journal.pone.0025057

**Published:** 2011-09-22

**Authors:** Ewa Jaworowska, Joanna Trubicka, Marcin R. Lener, Bartłomiej Masojć, Elżbieta Złowocka-Perłowska, James D. McKay, Hélène Renard, Dorota Oszutowska, Dominika Wokołorczyk, Jakub Lubiński, Tomasz Grodzki, Piotr Serwatowski, Katarzyna Nej-Wołosiak, Aleksandra Tołoczko-Grabarek, Andrzej Sikorski, Marcin Słojewski, Anna Jakubowska, Cezary Cybulski, Jan Lubiński, Rodney J. Scott

**Affiliations:** 1 Department of Otolaryngology and Laryngological Oncology, Pomeranian Medical University, Szczecin, Poland; 2 International Hereditary Cancer Center, Department of Genetics and Pathomorphology, Pomeranian Medical University, Szczecin, Poland; 3 Department of Radiotherapy, Western Pomeranian Oncology Center, Szczecin, Poland; 4 International Agency for Research on Cancer (IARC), Department of Genetic Epidemiology, Lyon, France; 5 Regional Hospital for Lung Diseases, Szczecin-Zdunowo, Poland; 6 Department of Urology and Urological Oncology, Pomeranian Medical University, Szczecin, Poland; 7 Discipline of Medical Genetics, University of Newcastle and The Hunter Medical Research Institute, Newcastle, New South Wales, Australia; Ohio State University Medical Center, United States of America

## Abstract

Genetic factors associated with the risk of smoking related cancers have until recently remained elusive. Since the publication of a genome-wide association study (GWAS) on lung cancer new genetic loci have been identified that appear to be associated with disease risk. In this replication study we genotyped 14 single nucleotide polymorphisms (SNPs) located at the 5p12.3-p15.33, 6p21.3-p22.1, 6q23-q27 and 15q25.1 loci in 874 lung, 450 bladder, 418 laryngeal cancer cases and cancer-free controls, matched by year of birth and sex to the cases. Our results revealed that loci in the chromosome region 15q25.1 (rs16969968[A], rs8034191[G]) and 5p15 (rs402710[T]) are associated with lung cancer risk in the Polish population (smoking status adjusted OR = 1.45, 1.35, 0.77; p≤0.0001, 0.0005, 0.002; 95%CI 1.23–1.72, 1.14–1.59, 0.66–0.91 respectively). None of the other regions analyzed herein were implicated in the risk of lung, bladder or laryngeal cancer. This study supports previous findings on lung cancer but fails to show association of SNPs located in 15q25.1 and 5p15 region with other smoking related cancers like bladder and laryngeal cancer.

## Introduction

Lung cancer remains the leading cause of cancer death. Every year more than 1 million new cases are diagnosed and a significant proportion die within two years of diagnosis [1]. Tobacco smoking is the major risk factor for lung cancer, but there is a distinct group of patients who develop the disease without a history of tobacco smoking. Furthermore, there are reports suggesting that a positive family history of lung cancer is an important risk factor for this disease [2].

Despite a large number of studies aimed at identifying genetic factors that modify lung cancer risk, no clear picture has emerged. In 2008 genome wide association studies (GWAS) revealed a series of single-nucleotide polymorphisms (SNPs) occurring at distinct loci, strongly associated with various aspects of disease risk. A locus at chromosome 15q25.1 was associated with lung cancer risk [3]–[9], nicotine and alcohol dependence [10]–[14]. Polymorphic markers of this particular region were located in the region of the nicotinic acetylcholine receptor gene *CHRNA5*, *CHRNA3* and the aminoglycoside phosphotransferase domain containing 1 gene *AGPHD1*. There is also evidence that other loci are associated with lung cancer risk: 5p15, which contains the TERT gene and the CLPTM1L gene [15]–[19]; 6p22.1, encompassing the MHC region [3], [15]; 6p21.33 which includes a region where the MSH5 gene (rs3131379) resides [3], [15], [19]; and 6q23-25 locus harboring the RGS17 gene [20]–[22].

To replicate and extend the association identified by the GWASs in lung cancer, we chose a series of markers from the loci 5p12.3-15.33, 6p21.33-22.1, 6q23-27 and 15q25.1, and examined their association with other smoking related neoplasms. A total of 874 lung, 418 laryngeal, 450 bladder cancer patients were sex and age matched with controls and assayed for 14 SNPs that are located in the four regions of interest (Table 1).

**Table 1:** Short Nucleotide Polymorphisms (SNPs) analysed in the study.

region	rs#	gene name	localization	Reference Allele	Rare Allel
5p15.33	rs2736100	telomerase reverse transcriptase (TERT)	intronic	T	G
	rs2736098	telomerase reverse transcriptase (TERT)	A [Ala] ⇒ A [Ala]	G	A
	rs402710	CLPTM1-like (CLPTM1L)	intronic	C**	T
5p14	rs17374971*	not located in gene	----	T	C
5q12.3	rs1387630	not located in gene	----	T	C
6p22.1	rs4324798	not located in gene	----	G	A
6p21.3	rs3131379	mutS homolog 5 (E. coli) (MSH5)	intronic	C	T
6q23	rs1545092	not located in gene	----	T	C
6q24.2	rs4286803	not located in gene	----	T	C
6q24.3	rs1007475	not located in gene	----	A	C
6q25	rs671330	not located in gene	----	G	A
6q27	rs7452888	not located in gene	----	A	G
15q25	rs8034191	aminoglycoside phosphotransferase domain containing 1 (AGPHD1)	intronic	T	C
	rs16969968	cholinergic receptor, nicotinic, alpha 5 (CHRNA5)	D [Asp] ⇒ N [Asn]	G	A

*not in Hardy Weinberg Equilibrium in one of control groups, excluded from further analysis.

**Note that in other studies minor allele (T) was the reference allele.

## Results

Out of 14 SNPs genotyped, one (rs17374971) was excluded from further analysis in all groups due to a statistically significant deviation from Hardy-Weinberg equilibrium.

Two SNPs on chromosome 15q25 (rs16969968 and rs8034191) were strongly associated with lung cancer (p<0.0001, p = 0.0002, respectively, Table 2) and were in strong linkage disequilibrium (D′ = 0.958, r^2^ = 0.91). Due to the high LD between rs16969968 and rs8034191, only rs16969968 will be discussed further. The odds ratio (OR) and 95% confidence interval (95% CI) for being heterozygous for rs16969968 when adjusted for smoking status was 1.68 (95% CI: 1.31–2.14) and when homozygous was 1.89 (95% CI: 1.32–2.70); when incorporated into the log by additive genetic effects model the OR was 1.45 (95% CI: 1.23–1.72). Although we observed the strongest association of rs16969968 and rs8034191 with the squamous cell carcinoma subtype of lung cancer (adjusted OR = 1.4 and 1.3; p = 0.002 and 0.017, respectively), a statistically significant association with small cell carcinoma subtype (adjusted OR = 1.5, 1.4; p = 0.017, 0.025) and a tendency towards an association with the adenocarcinoma subtype were observed (Table S1). No association between rs16969968 and rs8034191 with the age of lung cancer diagnosis, sex or smoking status could be identified (data not shown). Neither of these SNPs was associated with bladder or laryngeal cancer risk (Table S2 and S3). In a combined analysis of all control groups, the frequency of rs16969968 and rs8034191 was similar in the ever smokers compared to the never smokers group (adjusted for age and sex OR = 0.96, 0.97; p = 0.74, p = 0.8, respectively).

**Table 2:** Risk estimates for rs16969968, rs803419 and rs402710 in lung cancer group.

			Lung cancer		unadjusted results				results adjusted for smoking status			
rs#			Cases	Controls	OR	p-value	95% Conf. Interval		OR	p-value	95% Conf. Interval	
rs16969968	allel	G	993 (59,0%)	1115 (66,3%)	1,0	—	—	—	—	—	—	—
		A	691 (41,0%)	567 (33,7%)	1,38	<0,0001	1,20	1,59	1,45	<0,0001	1,23	1,72
	genotype	GG	280 (33,3%)	373 (44,4%)	1	—	—	—	—	—	—	—
		GA	433 (51,4%)	369 (43,9%)	1,56	<0,0001	1,27	1,92	1,68	<0,0001	1,31	2,14
		AA	129 (15,3%)	99 (11,8%)	1,74	0,0004	1,28	2,35	1,89	0,0005	1,32	2,70
rs8034191	allel	T	991 (59,5%)	1097 (66,0%)	1,0	—	—	—	—	—	—	—
		C	675 (40,5%)	565 (34,0%)	1,33	0,0002	1,15	1,53	1,35	0,0005	1,14	1,59
	genotype	TT	286 (34,3%)	368 (44,3%)	1,00	—	—	—	—	—	—	—
		TC	419 (50,3%)	361 (43,4%)	1,49	0,0002	1,21	1,84	1,56	0,0004	1,22	2,00
		CC	128 (15,4%)	102 (12,3%)	1,61	0,0018	1,19	2,19	1,63	0,0076	1,14	2,33
rs402710	allel	C*	1153 (68,0%)	1071 (63,4%)	1,0	—	—	—	—	—	—	—
		T	543 (32,0%)	619 (36,6%)	0,82	0,005	0,71	0,94	0,77	0,0020	0,66	0,91
	genotype	CC	398 (46,9%)	342 (40,5%)	1,00	—	—	—	—	—	—	—
		CT	357 (42,1%)	387 (45,8%)	0,79	0,026	0,65	0,97	0,75	0,021	0,59	0,96
		TT	93 (11,0%)	116 (13,7%)	0,69	0,018	0,51	0,94	0,61	0,007	0,43	0,87

*Note that in other studies minor allele (T) was the reference allele.

The association with lung cancer was also observed for the rs402710 SNP located on chromosome 5p15.33. This SNP, was in linkage disequilibrium with rs2736098 (D′ = 0.89; r^2^ = 0.129) but not with rs2736100 (D′ = 0.181; r^2^ = 0.014). The adjusted OR for carrying rare allele (T) of rs402710 was 0.77 (95%CI 0.66–0.91; p = 0.002). Both heterozygotes (CT) and rare homozygotes (TT) were less frequent in the lung cancer group compared to matched controls (adjusted OR = 0.75, 0.61; p = 0.021, 0.007, respectively) (Table 2). Furthermore, an association of rs402710 with lung squamous cell carcinoma and adenocarcioma subtypes was observed (adjusted OR = 0.69, 0.75; p = 0.001, 0.033, respectively) (Table S1). Similar to the observations related to the SNPs on chromosome 15q25.1, no statistically significant difference was detected when the lung cancer cases were stratified by sex, age of diagnosis and smoking status (data not shown). In a combined analysis of all control groups, rs402710 was not associated with smoking status (OR adjusted for sex and age of diagnosis = 1.03; p = 0.66).

Initially another association of lung cancer and rs3131379 (unadjusted OR = 1.47, p = 0.003) was observed, but after adjustment for smoking status it became statistically insignificant (Table S2 and S3). No associations of lung cancer with the remaining 10 SNPs genotyped were observed in this study (Table S2 and S3).

Analysis of 13 polymorphic markers among laryngeal and bladder cases revealed that none of them were associated with these cancer types (Table S2 and S3).

Retrospective power calculations were performed with Minor Allele Frequencies (MAF) ranging from 0.06–0.48 to ensure that the studied was sufficiently powered to identify any associations. This study had 80% power at an alpha level = 0.05 to detect minimum odds ratios in the range of 1.3–1.5 for lung cancer, 1.5–1.8 for bladder cancer and laryngeal cancer. The detection limit increased to 1.4–1.7 (lung cancer) and 1.7–2.1 (bladder and laryngeal cancer) with Bonferroni correction (alpha level = 0.0038).

## Discussion

This replication study demonstrated that SNPs in the chromosome 15q25.1 (rs16969968 and rs8034191) and 5p15 (rs402710) are associated with lung cancer in the Polish population thereby replicating previous findings [3]–[9], [15]–[19]. It did not, however, confirm any association between SNPs located in 15q25.1 and 5p15 region with bladder and laryngeal cancer.

The locus on chromosome 15q25.1 contains three genes that encode nicotinic acetylcholine receptor (nAChR) subunits (CHRNA3, CHRNA5, CHRNB4) whose involvement in tobacco addiction was suggested from studies conducted on larger lung cancer populations [5], [10]–[12]. The region of association contains the non-synonymous CHRNA5 SNP, rs16969968, which introduces a substitution of aspartic acid (D) for asparagine (N) at amino acid position 398 (D398N) of the CHRNA5 protein (α5 nAChR subunit). The α5 nAChR subunit is located in the cytoplasmic loop between the transmembrane domains and is not involved in receptor binding. The region of the CHRNA5 protein where the aspartic acid substitution at position 398 occurs is highly conserved across vertebrate species, suggesting that changes at this position are very likely to be of functional significance. *In vitro* studies indicate that aspargine at postion 398 of the α5 nAChR subunit decreases cholinergic receptor function [13]. The nicotinic receptors containing α5 are found in dopaminergic and GABAergic neurons in the striatum and ventral tegmental area, a region of the brain implicated in the reward pathway [13], [23]. Individuals who harbour the variant of CHRNA5 (which decreases cholinergic receptor activity) may have an increased risk of nicotine dependence as higher levels of nicotine are required to achieve similar activation of the dopaminergic pathway [13]. Once exposed to smoking, heterozygotes and rare homozygotes of rs16969968 have respectively a 1.3-fold and almost 2-fold increased risk of developing nicotine dependence [24]. Independent observations confirmed the association of SNPs in this region with the risk of tobacco related neoplasms like lung, bladder and upper aeordigestive tract cancers (UADT) [3]–[9], [25]–[27]. In support of these findings other studies have shown that this locus is implicated in the risk of chronic obstructive pulmonary disease (COPD) and peripheral arterial disease, both of which have been associated with smoking [5], [28]. Taken together these observations raise the question as to whether the 15q25.1 region is associated with a direct effect on smoking related diseases or can they be explained solely by a genetic influence on smoking addiction. Additional studies conducted on different populations supported the association of the 15q25.1 locus with lung cancer in never smokers providing evidence of a direct role of this locus in disease development [3], [6]. In support of this, it has been shown that pulmonary neuroendocrine cells, alveolar epithelial cells, pulmonary neuroendocrine cells and lung cancer cell lines, express nicotine receptors, which bind substances of carcinogenic potential that include *N*′-nitrosonornicotine and nitrosamines. Thus carcinogens may promote neoplastic transformation by stimulating tumor growth and angiogenesis [29], [30]. Moreover, the results of a recently conducted study on CHRNA5 activity modulation in bronchial cells and in lung cancer cell lines suggested a potential influence of CHRNA5 on cell adhesion as well as cell motility and the regulation of p63, a homologous protein to the tumour suppressor p53 [31]. Studies in large populations of mixed ethnicity showed that this locus is implicated in all histopathologic subtypes of lung cancer, in the current study we also replicated this association in the Polish population, although our analysis of risk alleles among the adenocarcinoma subtype cases showed only a weak tendency towards an association (Table S1) [3]–[9]. We were unable to show any association between SNPs in the 15q25 region and smoking status among controls, whereas other larger studies analyzing only nicotine dependence and concerning different measures of smoking exposure, clearly proved this association [10]–[13]. Our results were based on smaller numbers of never smoking lung cancer patients and we therefore lacked any power to detect this association. Our findings were, nevertheless, in agreement with a large replication study conducted within the International Lung Cancer Consortium using 11 645 lung cancer case patients and 14 954 control subjects, where no association of rs16969968, rs8034191 with lung cancer among never smokers was observed [9]. Adding to this, a recently published meta analysis of 5 previous studies on never smoking lung cancer patients found no association between 15q25 and lung cancer risk [32]. This puts into question the pleiotropic notion of the 15q25 region and suggests a more indirect effect on lung cancer risk.

The association of bladder cancer with the 15q25 locus was demonstrated in a case-control study of patients from Los Angeles County, USA and Shanghai, China. In this study rs8034191 was associated with 1.26-fold increased risk of bladder cancer among Non-Hispanic Whites, although the authors could not replicate the association in the Chinese population because the rare allele frequency was too low [25]. In contrast, our data and that from a study of 790 ever smoking bladder and renal cancer cases of Caucasian origin indicated no association between bladder cancer and the 15q25 region [33].

The region on chromosome 5p15.33 contains two genes, *CLPTM1L* - cleft lip and palate transmembrane 1 like gene and *TERT* - human telomerase reverse transcriptase gene. The TERT enzyme is a protein component of telomerase, a ribonucleoprotein polymerase that regenerates telomere ends by the addition of nucleotide repeat sequences. The RNA component of telomerase serves as a template for the telomere repeat. Telomerase plays a crucial role in securing chromosome stability and preventing normal cells becoming malignant. The coding region of TERT is highly conserved between species [34]. Mutations in the coding sequence of this gene are rare and have been associated with dyskeratosis congenita, idiopathic pulmonary fibrosis and an increased risk of some cancers [35], [36].

The second gene in 5p15.33 is *CLPTM1L*, which is implicated in the susceptibility to cleft palate. This gene is expressed in various tissues, including lung tissue and overexpressed in cisplatin-resistant ovarian cancer cell lines, where its role in the induction of apoptosis in cisplatin-sensitive cells has been demonstrated [37]. Some authors suggest that on the basis of these observations *CLPTM1L* could be associated with apoptosis induction in lung cells after exposure to genotoxic agents such as tobacco carcinogens [16].

In this study we have investigated two SNPs (rs2736100, rs2736098) that are situated in the region of TERT and in the region of CLPTM1L (rs402710). Currently, there is no evidence to suggest that any of those SNPs have a functional role or is a disease causative allele. The region 5p15.33 has been clearly associated with lung cancer [15]–[19]. In this study we replicated the association of rs402710 with lung cancer risk in the Polish population, but were unable to show an association of two other SNPs, one of which (rs2736100) was suggested to be an independent risk factor for lung cancer [16]. Stratification of our data by histopathological subtype revealed that the strongest association was observed among squamous cell carcinoma patients and a weaker association for the adenocarcinoma subtype, which is in contrast to results obtained from a GWAS, where the reverse relationship was observed [17], [18]. The 5p15.33 region was associated with bladder cancer in a study conducted by deCODE Genetics on a European population consisting of 4,147 cancer cases from 10 countries or regions [38]. In this large study rs401681 and rs2736098 were associated with an increased risk of bladder cancer (OR = 1.12, 1.16, p = 5.7×10^−5^, 1.3×10^−4^, 5.7×10^−5^, respectively). A study conducted on Non-Hispanic Whites from Los Angeles county, USA and a Chinese population showed a statistically significant association of bladder cancer with rs2736100 in both ethnic groups [25]. In our dataset we could not show the association of the three SNPs in the 5p15.33 region, probably due to population differences, which was also seen in the study by deCODE Genetics, where the rs2736098 association when stratified by country or region, reached a p value≤0.05 in only four of the contributing countries and regions [38].

A retrospective power analysis performed in our study suggested that one of the possible reasons we could not replicated the findings previously reported for bladder and laryngeal cancer groups could be due to the small sample size of these groups in our study. This problem could be addressed by increasing the case to control ratio from 1∶1 to 1∶3. In this study we were unable to match cases and controls with higher ratios due to not being able to recruit more controls that adhered to our matching criteria.

In summary this study has further confirmed the GWAS finding that the regions 15q25.1 and 5p15.33 contribute to lung cancer risk, but failed to show an association of these loci with bladder and laryngeal cancer in the Polish population.

## Materials and Methods

Between the years 2000 and 2007 a total of 1742 consecutively collected cancer patients from clinical hospitals in Szczecin, Poland were included in this study. The study comprised 874 cases of lung cancer, 450 patients diagnosed with bladder cancer and 418 laryngeal cancer patients. All cases were histologically or cytologically confirmed. Patients were invited to participate in this study when they attended an outpatient oncology clinic in their respective regional hospitals or the International Hereditary Cancer Center (IHCC) in Szczecin. The patient's participation rate exceeded 80%. Data on smoking status was available from 91% of all cancer patients, collected at the time of registration. For those patients where smoking status was not collected at the time of registration it was acquired either at follow-up visits or by personal communication (telephone, letter). Smoking status was categorized into two categories: never and ever smokers.

The control population consisted of 1061 healthy adult patients who had visited their family doctors located in the city of Szczecin or surrounding counties. The participation rates for the control population exceeded 71%. Cases and Controls were then randomly matched by year of birth (+/−3 years) and sex, resulting in 874, 450, 418 pairs with lung, bladder and laryngeal cancer cases, respectively. Data on smoking status was available from 83% of all controls. The characteristics of cases and control groups are presented in the Table 3.

**Table 3:** Details of cancer patients and healthy controls.

	Lung cancer		Bladder cancer		Laryngeal cancer	
	Cases	Controls	Cases	Controls	Cases	Controls
Number	874	874	450	450	418	418
Males (%)	644 (73,7%)	644 (73,7%)	346 (76,9%)	346 (76,9%)	348 (83,3%)	348 (83,3%)
Females(%)	230 (26,3%)	230 (26,3%)	104 (23,1%)	104 (23,1%)	70 (16,7%)	70 (16,7%)
Mean year of birth (range)	1943 (1916–1976)	1943 (1915–1976)	1940 (1916–1979)	1940 (1915–1976)	1944 (1919–1971)	1944 (1920–1971)
Mean Age (range)	61 (28–88)	61 (28–88)	63 (25–88)	63 (28–88)	58 (30–84)	58 (32–81)
Smoking status						
neversmokers (%)	44 (5,0%)	249 (28,5%)	55 (12,2%)	156 (34,7%)	10 (2,4%)	83 (19,9%)
eversmokers (%)	765 (87,5%)	436 (49,9%)	333 (74,0%)	228 (50,7%)	378 (90,4%)	313 (74,9%)
missing data	65 (7,4%)	189 (21,6%)	62 (13,8%)	66 (14,7%)	30 (7,2%)	22 (5,3%)
Histopathology classification						
squamous cell*/papillary urothelial**/squamous cell***	328 (37,5%)	-	303 (67,3%)	-	416 (99,5%)	-
small cell*/invasive urothelial**/carcinosarcoma***	100 (11,4%)	-	104 (23,1%)	-	2 (0,5%)	-
adenocarcinoma*/urothelial carcinoma (NOS)**	184 (21,1%)	-	40 (8,9%)	-	-	-
large cell*/adenocarcinoma**	33 (3,8%)	-	1 (0,2%)	-	-	-
adenosqaumous*/other**	4 (0,5%)	-	2 (0,4%)	-	-	-
carcinomas with pleomorphic, sarcomatoid or sarcomatous elements*	4 (0,5%)	-	-	-	-	-
carcinoid tumour*	1 (0,1%)	-	-	-	-	-
carcinoma of salivary-gland type*	2 (0,2%)	-	-	-	-	-
non-small-cell carcinoma (not otherwise specified by pathologist)*	149 (17,0%)	-	-	-	-	-
other* #	69 (7,9%)	-	-	-	-	-
Histopathology by clinical classification						
non-small-cell carcinoma*	704 (80,5%)	-	-	-	-	-
small-cell carcinoma*	100 (11,4%)	-	-	-	-	-
other* #	70 (8,0%)	-	-	-	-	-

*lung cancer histopathology.

**bladder cancer histopathology.

***laryngeal cancer histopathology.

# subtype not in classification, identified by pathologist in Fine Needle Aspiration biopsy as “cellulae carcinomatose”.

The study was approved by Ethics Committee of the Pomeranian Medical University of Szczecin. All individuals included in this study were only enrolled after providing written informed consent to participate.

The DNA for analysis was extracted from peripheral blood lymphocytes of individuals using the non-enzymatic, rapid salting-out method without modification as described previously [39]. The genotyping analysis of 14 SNP was performed using the 5′exonuclease assay (TaqMan, Applied Biosystems, Foster City, CA, U.S.A) at the International Agency for Research on Cancer. DNA samples from cancer and control cases were randomly allotted assay numbers during the genotyping process in order to avoid any genotyping bias; laboratory personnel were blinded to case/control status. 7% of the total number of subjects in this study (derived from both cases and controls) were randomly selected to be re-genotyped for each SNP to control for the reproducibility of the genotyping assays. Internal duplicate concordance was >99.9% and genotyping success rate was >91%. Out of 14 analysed SNPs, the genotype distributions of 13 SNPs did not deviate from Hardy-Weinberg Equilibrium (HWE) in any group of matched controls. One SNP (rs17374971) that had significant deviation from HWE was excluded from further analysis in all groups. Each of the SNPs was analysed individually per allele (under the log-additive model) and per genotype by calculating odds ratio (OR), 95% confidence interval (95% CI), p-values using a logistic regression model. OR adjustment was performed by incorporating smoking status as an additional parameter in logistic regression model. Patients with missing data on smoking status were excluded from the calculation of an adjusted OR. Alleles for all SNPs were assigned according to Single Nucleotide Polymorphism database (dbSNP, http://www.ncbi.nlm.nih.gov/SNP) [40]. All calculations were performed using the major allele of each SNP as the reference (Table 1). Bonferroni correction was used to adjust for multiple testing such that all results that obtained a p value≤0,0038 were considered significant. LD (D′ and r^2^) was calculated using haploview [41]. A retrospective power analysis was performed using Power for Genetic Association Version 2.0 software with the alpha level set at 0.05 and 0.0038 under co-dominant model with two degrees of freedom [42].

## Supporting Information

Table S1 Risk estimates per allele by histopathology for rs16969968, rs803419 and rs402710 in lung cancer group.
(XLS)

Table S2 Risk estimates per allele for 13 SNPs in lung, bladder and laryngeal cancer group.
(XLS)

Table S3 Risk estimates per genotype for 13 SNPs in lung, bladder and laryngeal cancer group.
(XLS)
